# Volumes of Velopharyngeal and Glossopharyngeal Airway Were Not Changed after Uvulopalatopharyngoplasty: Report of Three Cases

**DOI:** 10.1155/2016/9378428

**Published:** 2016-03-24

**Authors:** Yoichi Nishimura, Naoko Fujii, Takahisa Yamamoto, Mahmood A. Hamed, Misato Nishimura, Takuro Kojima, Noboru Iwata, Kenji Suzuki, Seiichi Nakata

**Affiliations:** ^1^Department of Otorhinolaryngology, Second Hospital, Fujita Health University School of Medicine, 3-6-10 Otobashi, Nagoya, Aichi 454-8509, Japan; ^2^Department of Radiology, Second Hospital, Fujita Health University School of Medicine, Nagoya, Aichi 454-8509, Japan; ^3^Department of Mechanical Engineering, Gifu National College of Technology, Motosu, Gifu 501-0400, Japan; ^4^Department of Otolaryngology, Sohag University, Sohag 82542, Egypt; ^5^Yonaha General Hospital, Kuwana, Mie 511-0000, Japan

## Abstract

*Objective*. The aim of this study was to investigate the changes in velopharyngeal and glossopharyngeal airway morphology and volume after uvulopalatopharyngoplasty in three adult obstructive sleep apnea syndrome patients who had bilateral large tonsils using three-dimensional computed tomography.* Case Report*. All three patients (one male and two females) who presented with a history of heavy snoring and excessive daytime sleepiness were examined with overnight nocturnal polysomnography, which indicated moderate-to-severe obstructive sleep apnea syndrome. Because all patients had large tonsils, uvulopalatopharyngoplasty was expected to enlarge the pharyngeal airway. Polysomnography and three-dimensional computed tomography scanning were performed and compared, both before and 3 months after uvulopalatopharyngoplasty.* Results*. Unexpectedly, although the morphology of the glossopharyngeal airway clearly changed after UPPP, the volume changes in the velopharyngeal and glossopharyngeal airways were negligible.

## 1. Introduction

Obstructive sleep apnea syndrome (OSAS) increases the risk of developing cardiac diseases and hypertension [1]. Therefore, effective treatment of patients with OSAS is critical. Recent imaging studies have described pharyngeal morphology in patients with OSAS. In particular, the assessment of upper airway volume has received much attention from specialists in OSAS as a challenging approach to understanding the pathogenesis of OSAS [2–4]. The occlusion of the upper airway in OSAS mainly affects the velopharyngeal and glossopharyngeal portions [5]. Uvulopalatopharyngoplasty (UPPP) is a surgical procedure that is used to enlarge the pharyngeal space. To the best of our knowledge, there is no report that has used 3D CT images to quantify the change in air volume in the velopharyngeal and glossopharyngeal airway from pre- to post-UPPP in adult OSAS patients.

In this report, we present velopharyngeal and glossopharyngeal morphology and volume before and after UPPP, assessed by three-dimensional computed tomography (3D CT) in three adult OSAS patients who had bilateral large tonsils.

## 2. Case Presentation

All three patients (one male and two females) who presented with a history of heavy snoring and excessive daytime sleepiness, indicated by a high Epworth Sleepiness Scale score, were referred to our department by a medical practitioner. They requested surgical treatment to enlarge their pharyngeal airway. Before surgical treatment, all three patients underwent a complete otorhinolaryngological physical examination that was conducted by a researcher. Body mass index, tonsil size, and palate grade were recorded for analysis. Body mass index was calculated as weight (kg)/height (m)^2^. Tonsil size and palate grade were evaluated according to the scale proposed by Friedman et al. [6]. All patients were examined with overnight nocturnal polysomnography in our sleep laboratory in the standard manner, and each had an apnea hypopnea index >20 events/hour, indicating moderate-to-severe OSAS.

Because the patients had bilateral large tonsils, the UPPP surgical procedure to enlarge the pharyngeal airway was performed by a sleep surgeon under general anesthesia, according to the originally described technique [7]. The large tonsils were removed bilaterally and the tonsil's weights were measured. All patients tolerated the surgery without complications.

Body mass index and Epworth Sleepiness Scale score were evaluated both before and 3 months after UPPP. Polysomnography and 3D CT scanning were also performed at both time points. Postoperative polysomnography revealed moderate-to-great improvement in the severity of OSAS (Table 1).

### 2.1. 3D CT

3D CT was performed under the same condition before and after UPPP. 3D CT was performed using a 64-row multidetector CT scanner (Brilliance 64®, Phillips, Cleveland, OH, USA) under routine neck examination conditions at the end of inspiration while the patients were holding their breath. Thin axial sections of 0.9 mm thickness at 0.45 mm intervals were obtained to reconstruct multiplanar reformation images and 3D CT images. Multiplanar reformation and 3D CT image reconstructions were performed at an image workstation (Ziostation2, Ziosoft, Tokyo, Japan), which enabled us to visualize and objectively quantify the dimensions and volume of the airway. Axial-view multiplanar reformation images were used for this analysis. The 3D CT images of the velopharyngeal and glossopharyngeal airway were constructed from images of areas in which the CT number was −400 Hounsfield units or less.

**Figure 1:**
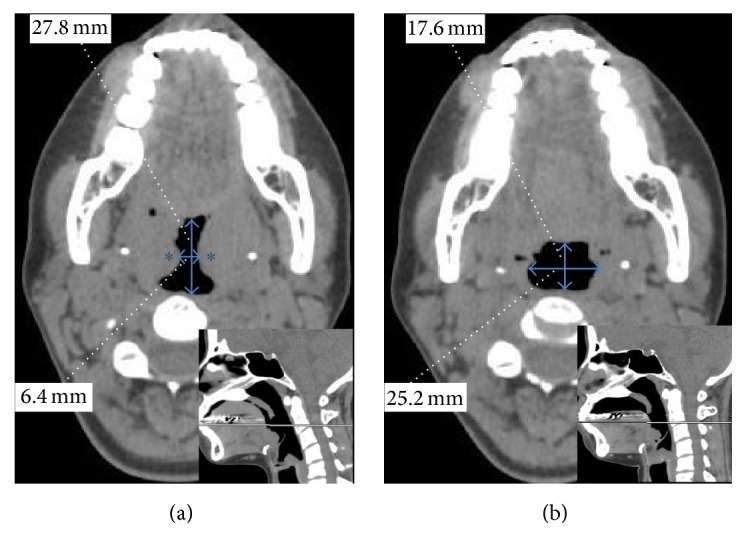
Axial-view computed tomography images from patient 1. (a) The narrowing of glossopharynx (blue double-headed arrows) evident before uvulopalatopharyngoplasty (UPPP) was caused by the impression of large tonsils (asterisk). (b) The narrowing was enlarged transversely; however, the glossopharynx became narrower in the anteroposterior direction after UPPP (indicated by the number). The lines on midsagittal view indicate the level of the axial-view.

Axial and sagittal images of the glossopharynx before (Figure 1(a)) and after (Figure 1(b)) UPPP in patient 1 were shown in Figure 1. The maximal diameter of the glossopharynx in the transverse dimension and the minimal diameter in the anteroposterior dimension were recorded for analysis.

**Figure 2:**
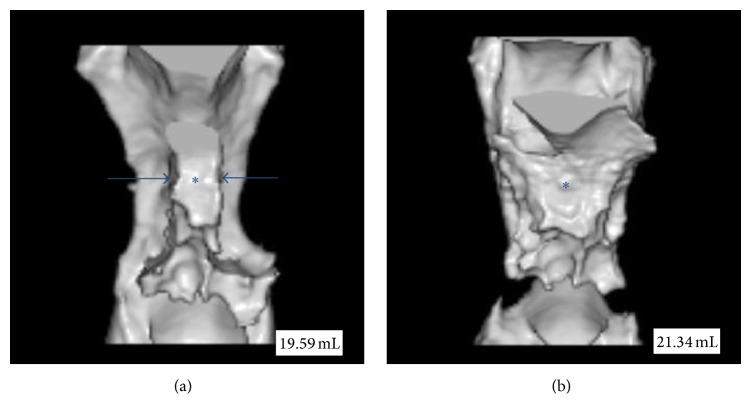
Anterior-view three-dimensional computed tomography images from patient 1. (a) Bilateral large tonsils impressed on the glossopharyngeal airway from both sides (blue arrows) before uvulopalatopharyngoplasty (UPPP). The gap between two arrows shows the airway (asterisk). (b) The airway was enlarged after UPPP. Nevertheless, the change in the volume of the airway (indicated by the number) was negligible.

**Figure 3:**
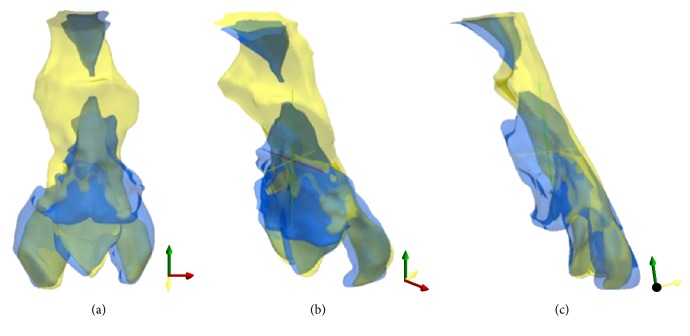
3D composite images before UPPP (blue) and after UPPP (yellow) from patient 1. Anterior view (a), left oblique view (b), and left lateral view (c). The 3D composite images were superimposed to visualize these morphological changes clearly.

In patient 1, the transverse diameter of the glossopharynx was enlarged from 6.4 mm preoperatively to 25.2 mm postoperatively, whereas the anteroposterior diameter was reduced from 27.8 mm to 17.6 mm. In like manner, in patients 2 and 3, the transverse diameter was enlarged, whereas the anteroposterior diameter was reduced postoperatively. To provide a better understanding of these morphological changes, the anterior-view 3D CT images of the velopharyngeal and glossopharyngeal airway in patient 1 before (Figure 2(a)) and after (Figure 2(b)) UPPP were shown in Figure 2. In addition, 3D composite images from before and after UPPP in patient 1 were also generated at an image workstation (ParaView®, Kitware, Clifton Park, NY, USA). 3D images were adjusted for their opacities and displayed by superimposition in Figure 3.

The following limits were adopted when measuring the velopharyngeal and glossopharyngeal airway volume: the superior margin was the posterior nasal spine, and the inferior and anterior margins were the hyoid bone in the midsagittal view. The dimensions of the glossopharynx and the volume of the velopharyngeal and glossopharyngeal airway before and after UPPP in each of the three patients were summarized in Table 2. In all patients, the transverse diameter of the glossopharynx increased after UPPP, whereas the anteroposterior diameter decreased. Thus, morphological changes were observed after UPPP; however, the change in total air volume was negligible (Table 2).

## 3. Discussion

Since Fujita et al. [7] first described UPPP as a surgical procedure for OSAS in 1981, it has remained the main surgical approach for the treatment of patients with OSAS. UPPP is designed to enlarge the airway lumen at the level of the velopharyngeal area and decrease the collapsibility of the pharyngeal walls. UPPP enlarges the upper airway by removing the redundant excessive distal palatal tissue while preserving the function of the proximal palatal musculature. However, the mechanism by which UPPP improves OSAS is still controversial. Moreover, the success rate of this surgery, which is based mainly on the measurement of respiratory parameters, was only around 50% in a long-term follow-up study [8].

Patients with more severe OSAS tend to have larger tonsils and a smaller airway volume [9]. OSAS is considered to be caused by an imbalance in the pharyngeal airway size that is determined by the anatomical balance between the large volume of contents (i.e., tonsils and surrounding soft tissues structures) and the small volume of the container, which is constrained by craniofacial bony structures. When this anatomical balance theory [10] is applied to UPPP, the residual air space should be smaller when the contents are larger. The aim of UPPP should be to reduce the content volume, which should result in an increase in the volume of the pharyngeal airway.

In this report, for all three patients, bilateral large tonsils were removed as a part of UPPP. For example, in patient 1, the weight of the bilateral large tonsils which were removed during UPPP was 14.5 g (7.6 g and 6.9 g for the right and left side, resp.). It was expected that the air volume of the velopharyngeal and glossopharyngeal spaces would increase by the equivalent amount (i.e., 14.5 g/14.5 mL) after UPPP.

Our results showed obvious morphological changes after UPPP; however, changes in velopharyngeal and glossopharyngeal air volume were negligible—from 19.59 mL to 21.34 mL. As shown in Figure 1, the transverse diameter of the glossopharynx was enlarged from 6.4 mm to 25.2 mm; however, the anteroposterior diameter narrowed from 27.8 mm to 17.6 mm because the tongue base was displaced from anterior to posterior after UPPP. It was supposed that, before UPPP, the bilateral large tonsils maintain the tongue base in a forward position. However, after bilateral large tonsils were removed, the tongue base moved backwards, thus, reducing the AP diameter of the airway. These morphological changes could underlie the lack of change in air volume in the velopharyngeal and glossopharyngeal airway after UPPP.

To the best of our knowledge, there is no report that has used 3D CT images to quantify the change in air volume in the velopharyngeal and glossopharyngeal airway from pre- to post-UPPP in adult OSAS patients with large tonsils. Recently, Chiffer et al. [2] reported volumetric magnetic resonance imaging analysis before and after transoral robotic surgery for OSAS patients. They reported total airway volume increased postoperatively, whereas the volumes of the soft palate, tongue, and total and retropalatal lateral pharyngeal walls decreased. Cossellu et al. [3] also reported morphological and volume changes in the upper airway using 3D CT during oral appliance in patients with OSAS. They showed an improvement of the total upper air volume in nine out of ten patients. To our knowledge, no reports discussed volume changes before and after UPPP.

Schwab et al. [11] already described that the airway caliber increased substantially after UPPP, with a large increase in the lateral airway dimension and a decrease in the thickness of the lateral pharyngeal walls. Langin et al. [12] also performed CT in patients with OSAS before and after UPPP and demonstrated an increased width of the fauces after surgery. Nevertheless, these studies evaluated morphological changes but did not quantify volume change after UPPP.

The patients reported here indicate that the mechanism by which UPPP improves OSAS cannot fully be explained using the anatomical balance theory.

In three cases, there was marked improvement in OSAS symptoms as well as AHI and ESS scores despite negligible changes in the airway volume after UPPP. We believe that this improvement could be attributed to three factors. First, muscle tone might have increased after UPPP as a result of scar formation on the palatopharyngeal wound, which could have contributed to reduced collapsibility of the pharyngeal walls during sleep. Second, the morphological change, that is, enlargement of the restricted area, might have altered pharyngeal air circulation and swirl flow, which would be expected to normalize pharyngeal air flow during sleep. Finally, this postoperative alteration in anatomy could have led to an equalization of the airway lumen diameter. We believe this equalization implies a decrease in airflow velocity and postoperative diminution in the collapsibility of the pharyngeal air lumen during sleep according to Bernoulli effect [13]. However, these aeromechanics theories require further studies with more patients using computational fluid dynamics.

In this report, we presented small number of patients. However, our investigation showed that UPPP did not always increase air volume in the velopharyngeal and glossopharyngeal airway even after bilateral large tonsils were removed. This potentially valuable report suggests an alternative working mechanism of UPPP.

## 4. Conclusion

In conclusion, in our patients, the morphology of the velopharyngeal and glossopharyngeal airway changed after UPPP; however, the volume did not change. Further analysis of 3D CT images could contribute to our understanding of changes in morphology and air volume in the upper airway that are caused by UPPP.

## Figures and Tables

**Table 1:** Physiologic and polysomnographic variables before and after uvulopalatopharyngoplasty.

Patient	Age/sex	BMI (kg/m^2^)		Tonsils				ESS		AHI(events/h)		CT90(%)		LSAT(%)	
				TS	TW		PG								
		Pre-	Post-		R	L		Pre-	Post-	Pre-	Post-	Pre-	Post-	Pre-	Post-
1	36 F	34.9	31.0	3	7.6	6.9	IV	16	7	112.1	3.8	45.1	0.2	61	84
2	28 M	26.4	25.8	3	9.4	4.4	II	15	14	47.0	16.2	3.5	6.2	77	78
3	30 F	20.0	19.9	3	10.7	10.7	IV	13	5	22.9	0.4	0	0	94	95

AHI = apnea hypopnea index; BMI = body mass index; CT90 = percentage of time with oxygen saturation below 90%; ESS = Epworth Sleepiness Scale; F = female; M = male; pre- = preoperatively; post- = postoperatively; PG = palate grade; R = right; TS = tonsil size; TW = tonsil weight.

**Table 2:** Anatomical measures from 3D CT before and after uvulopalatopharyngoplasty.

Patient		3D CT			
		Diameter (mm)		Air volume (mL)	
		Pre-	Post-	Pre -	Post-
1	T	6.4	25.2	19.59	21.34
	A-P	27.8	17.6		

2	T	13.2	19.3	8.78	9.03
	A-P	14.8	12.1		

3	T	12.8	19.0	10.39	11.53
	A-P	16.8	12.6		

3D CT = three-dimension computed tomography; A-P = anterior-posterior; OSAS = obstructive sleep apnea syndrome; pre- = preoperatively; post- = postoperatively; T = transverse.

## Additional Points

This work was done in the Department of Otorhinolaryngology, Second Hospital, Fujita Health University School of Medicine, Nagoya, Aichi, Japan.
